# Progression of immunoglobulin A nephropathy (IgAN) in a Hispanic/Latinx population in the United States

**DOI:** 10.3389/fneph.2025.1744454

**Published:** 2026-01-09

**Authors:** John J. Sim, Nancy T. Cannizzaro, Qiaoling Chen, Sasikiran Nunna, Mohit Mathur, Cibele Pinto

**Affiliations:** 1 Division of Nephrology and Hypertension, Kaiser Permanente Los Angeles Medical Center, Los Angeles, CA, United States; 2 Department of Research and Evaluation, Kaiser Permanente Southern California, Pasadena, CA, United States; 3 Department of Clinical Science, Kaiser Permanente Bernard J. Tyson School of Medicine, Pasadena, CA, United States; 4 Otsuka Pharmaceutical Development and Commercialization, Inc., Rockville, MD, United States; 5 Visterra, Inc., Waltham, MA, United States

**Keywords:** epidemiology, IgAN disease progression, kidney failure (KF), Latinx (Hispanic), race ethnicity

## Abstract

**Background:**

Immunoglobulin A nephropathy (IgAN) is a leading cause of chronic kidney disease (CKD) worldwide. While racial and ethnic differences in disease progression are well documented, the Hispanic/Latinx populations remain understudied despite their elevated risk of kidney failure among other CKD populations.

**Objective:**

This study aimed to evaluate the kidney function decline and progression in Hispanic/Latinx patients with biopsy-proven IgAN within a large, integrated healthcare system and to contextualize to other racial/ethnic groups.

**Methods:**

We conducted a retrospective case series study of 259 Hispanic/Latinx adults with biopsy-proven IgAN from the Kaiser Permanente Southern California (KPSC) health system. Patients were followed from biopsy to ≥50% decline in the estimated glomerular filtration rate (eGFR), kidney failure, mortality, the study end date of November 30, 2022, or disenrollment. Annualized eGFR decline and the incidence of composite kidney outcomes were assessed.

**Results:**

At diagnosis, Hispanic/Latinx patients had significant CKD and a high risk of progression to kidney failure, indicated by a median eGFR of 56 ml min^−1^ 1.73 m^−2^ and a median urine protein/creatinine ratio of 1.8 g/g. Common treatments included immunosuppressive agents (41%), angiotensin-converting enzyme (ACE) inhibitors (48%), and angiotensin receptor blockers (ARBs; 20%). The mean annual eGFR decline was −4.5 ml min^−1^ 1.73 m^−2^, and 30.9% experienced rapid decline (>5 ml min^−1^ 1.73 m^−2^ per year). The composite kidney outcome occurred at 73.3 events per 1,000 patient-years, with a median time to event of 2.8 years and a median age at event of 46 years.

**Conclusion:**

Hispanic/Latinx patients with IgAN demonstrate rapid kidney function decline and early-onset kidney failure. These findings underscore the need for earlier detection and targeted management in this underserved group.

## Introduction

Immunoglobulin A nephropathy (IgAN) is one of the most common glomerular diseases and is a major cause of chronic kidney disease (CKD) (1). In contrast to earlier perceptions about an indolent disease course, studies are now showing that patients with IgAN have an elevated risk of kidney failure within their lifetimes (2, 3). A recent cohort study, for example, found that half of affected individuals reached an endpoint of kidney failure or mortality within 6 years of IgAN diagnosis, with nearly all expected to progress to kidney failure during their lifetimes (4). The epidemiology of IgAN varies by race/ethnicity, with populations of Asian ancestry exhibiting a higher incidence relative to European populations, who in turn have a higher risk than African populations (5). The natural history of IgAN also differs by race/ethnicity, with an elevated progression risk among Asian patients (4, 6).

Relatively few studies have been conducted to evaluate the disease incidence or the progression risk in Hispanic/Latinx populations, resulting in a gap in the understanding of IgAN in Latin America and among Hispanic/Latinx patients in the United States (US) (5). The available data indicate that IgAN is one of the most common glomerulopathies among Hispanics/Latinx people and that the prevalence of kidney failure due to IgAN has significantly increased in this group (7, 8). The Hispanic/Latinx population is the largest racial/ethnic minority in the United States and is at increased risk of kidney failure compared with non-Hispanic whites (9). However, there has been extremely low to no participation of Hispanic/Latinx patients in IgAN clinical trials. A recent retrospective, longitudinal cohort study of the progression of IgAN in the racially diverse membership of the Kaiser Permanente Southern California (KPSC) health system, which enrolled over 4.8 million individuals, assessed the outcomes among different racial groups (i.e., Asian/Pacific Islander, Black, Hispanic/Latinx, and white) (10, 11). Hispanic/Latinx patients exhibited the numerically highest incidence rates for kidney failure (60.8 *vs*. 13.8–60.3 events per 1,000 patient-years) and the youngest median age at kidney failure (46.1 *vs*. 47.1–73.1 years) relative to the other groups (11). Better understanding of the disease progression is clearly needed and could help improve the outcomes in the large and growing US Hispanic/Latinx population. To address this need, we conducted a secondary analysis of our KPSC IgAN cohort to determine the progression of decline in kidney function among Hispanic/Latinx KPSC patients with IgAN and to contextualize the findings relative to other populations.

## Methods

This retrospective case series comprised adult (age >18 years) KPSC members with a diagnosis of primary IgAN based on kidney biopsies performed between January 1, 2000, and December 31, 2021. Continuous KPSC membership for at least 6 months (allowing for gaps of <45 days) before kidney biopsy was required to capture comorbidity data. The exclusion criteria were secondary IgAN and existing kidney failure defined as dialysis, kidney transplant, or an estimated glomerular filtration rate (eGFR) <15 ml min^−1^ 1.73 m^−2^ (12). This study was approved by the KPSC Institutional Review Board (no. 5815) and exempted from informed consent.

The demographic and clinical data were obtained from the KPSC electronic health records. The information on race in the electronic health record was ascertained from patient self-reporting or reporting by the healthcare provider. Nearly all Hispanic/Latinx members enrolled in KPSC identified themselves as Hispanic/Latinx White, with less than 1% of KPSC members identifying themselves as Hispanic/Latinx Black. The 2021 Chronic Kidney Disease Epidemiology Collaboration equation was used to calculate the eGFR, and only the outpatient eGFR values were used (13). The kidney biopsy data were obtained from the KPSC Pathology Database as previously described (12). For individuals who had multiple biopsies, the first biopsy result was used for inclusion and was assigned as the index date. Data on angiotensin-converting enzyme inhibitor (ACEI) or angiotensin receptor blocker (ARB) use were obtained in the 1-year period prior to biopsy, whereas data on immunosuppressive medication treatment were obtained within 4 weeks prior to and 1 year after biopsy. The immunosuppressive treatments of interest were prescriptions of at least 7 days for steroids, calcineurin inhibitors, and alkylating agents, among others (e.g., sirolimus, azathioprine, and mycophenolate), as has been reported (11).

Study follow-up was from the date of biopsy (the index date) to the onset of ≥50% decline in eGFR, kidney failure, mortality, disenrollment, or the censoring date of November 30, 2022, whichever came first. The outcomes assessed included the annualized rate of eGFR decline, calculated using an ordinary least squares regression method. The model was fitted to all eGFR readings including the baseline and follow-up beyond 90 days from the baseline for each patient. An additional outcome was the proportion of patients who experienced rapid annual eGFR decline, defined as >5 ml min^−1^ 1.73 m^−2^ per year (14). The incidence of a composite kidney outcome defined as ≥50% decline in eGFR from the baseline or reaching kidney failure was also evaluated. A ≥50% decline in eGFR was determined from three eGFR values: the index value, a first eGFR value after the index with ≥50% decline from the index eGFR, and a confirmatory eGFR showing persistent ≥50% decline at ≥30 days from the date of the initial ≥50% decline. Kidney failure was defined as receiving dialysis, kidney transplantation, or reaching eGFR <15 ml min^−1^ m^−2^ as defined by two subsequent eGFR reports <15 ml min^−1^ m^−2^ at least 30 days apart.

Categorical variables were presented as frequencies and percentages, with chi-squared or Fisher’s exact tests conducted for comparison as appropriate. Continuous variables were reported as the mean (standard deviation, SD) or the median (interquartile range, IQR) and were compared using the Kruskal–Wallis test. The exploratory statistical comparisons of Hispanic/Latinx patients with other racial and ethnic groups were conducted to contextualize the findings for the Hispanic/Latinx population. The incidence of a composite kidney outcome was calculated per 1,000 patient-years. Poisson regression with robust standard error was used to calculate the 95% confidence intervals (CIs). Kaplan–Meier curves were plotted stratified by race/ethnicity, with *p*-values based on the log-rank test reported. Statistical analyses were conducted using SAS software (version 9.4; SAS Institute, Inc., Cary, NC, USA). Results with *p* < 0.05 were considered statistically significant.

## Results

**Figure 1:**
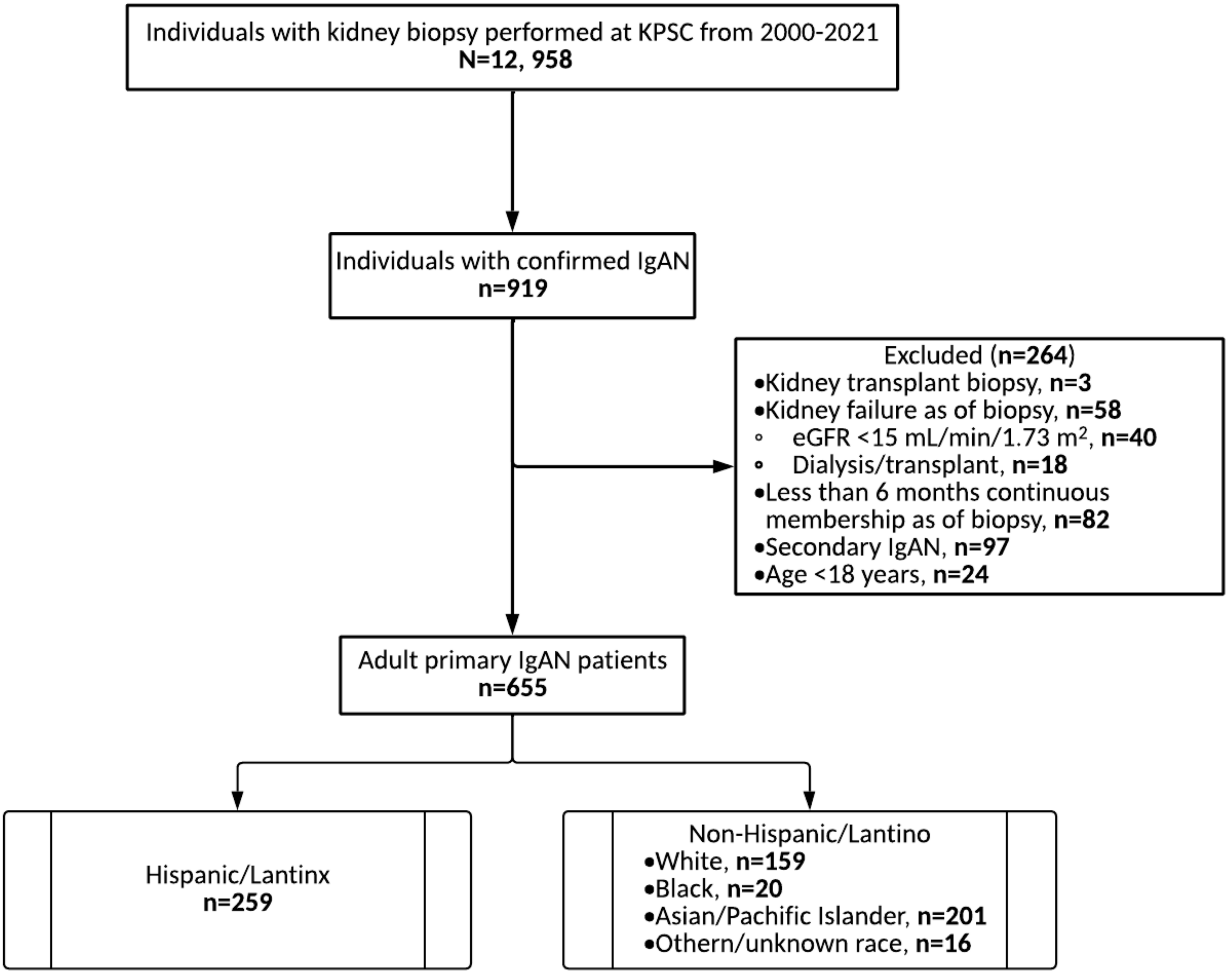
Identification of the study population. *eGFR*, estimated glomerular filtration rate; *IgAN*, immunoglobulin A nephropathy; *KPSC*, Kaiser Permanente Southern California.

Among 12,958 individuals with kidney biopsy performed from 2000 to 2021, 655 had IgAN confirmed and met the eligibility criteria for inclusion in the analysis, 259 (39.5%) of whom are Hispanic/Latinx (**Figure 1**). The mean (SD) age of the Hispanic patients with IgAN at the index date was 44.7 (14) years, with a nearly equal representation of women (49.4%) and men (50.6%) (**Supplementary Table S1**) (11). Hypertension (63.3%), hematuria (47.5%), and diabetes (14.3%) were the most frequently reported comorbidities. The median eGFR (IQR) value was 56.0 (36.6–82.8) ml min^−1^ 1.73 m^−2^, with a CKD stage distribution of: G1, 22%; G2, 24%; G3a, 16%; G3b, 25%; G4, 13%. For the urine protein/creatinine ratio (UPCR), a median (IQR) value of 1.8 (1.0–3.2) g/g was observed, with a UPCR distribution of: <0.5 g/g (12.4%); 0.5–1 g/g (10.4%); 1–2 g/g (28.6%); >2 g/g (42.9%), and unknown (5.8%). Among the Hispanic/Latinx patient cohort, 40.9% received immunosuppressive agents, 48.3% were on ACEIs, and 19.7% were on ARBs. Steroids comprised >90% of all immunosuppression medications prescribed. The kidney function parameters and the medication use at index among the Hispanic/Latinx patients did not exhibit any major differences from the other racial/ethnic groups studied (11).

The median (IQR) follow-up time was 3.3 years (1.4–6.8) for Hispanic/Latinx patients. Their mean (SD) annualized eGFR decline was −4.5 (10.5) ml min^−1^ 1.73 m^−2^, and their median (IQR) annualized decline was −3.0 (−6.6 to −0.6) ml min^−1^ 1.73 m^−2^ (**Table 1**). The rate of decline for the Hispanic/Latinx patients was closest to that for the Asian/Pacific Islander group, in which the mean annualized eGFR decline was −3.8 (10.3) ml min^−1^ 1.73 m^−2^ and the median (IQR) annualized decline was −2.4 (−6.6 to −0.4) ml min^−1^ 1.73 m^−2^. The Hispanic/Latinx group also had the numerically highest proportion of patients (30.9%) who met the threshold for rapid eGFR decline (>5 ml min^−1^ 1.73 m^−2^ per year). Although the differences among the groups did not reach statistical significance, it was observed that the rate of decline was highest among Hispanic/Latinx patients.

**Table 1:** Annual rate of decline in estimated glomerular filtration rate (eGFR) in biopsy-proven immunoglobulin A nephropathy (IgAN).

eGFR change	Asian/Pacific Islander (n = 201)	Black (n = 20)	Hispanic/Latinx (n = 259)	Other/Unknown (n = 16)	White (n = 159)	Overall P-Value
Annual eGFR change	eGFR change	eGFR change	eGFR change	eGFR change	eGFR change	0.15^a^
Mean (SD)	-3.8 (10.3)	-2.1 (13.9)	-4.5 (10.5)	1.5 (10.5)	-3.0 (15.4)	
Median (IQR)	-2.4 (-6.6, -0.4)	-0.9 (-5.1, 4.0)	-3.0 (-6.6, -0.6)	-0.8 (-4.6, 9.1)	-2.1 (-7.9, 0.1)	
Unknown	11	1	22	2	16	
Annual eGFR decline >5 mL/min/1.73 m^2^						0.79^b^
No	134 (66.7%)	14 (70.0%)	157 (60.6%)	11 (68.8%)	97 (61.0%)	
Yes	56 (27.9%)	5 (25.0%)	80 (30.9%)	3 (18.8%)	46 (28.9%)	
Unknown	11 (5.5%)	1 (5.0%)	22 (8.5%)	2 (12.5%)	16 (10.1%)	

*eGFR*, estimated glomerular filtration rate; *IQR*, interquartile range; *SD*, standard deviation.

a Kruskal–Wallis *p*-value.

b Fisher’s exact *p*-value.

**Figure 2:**
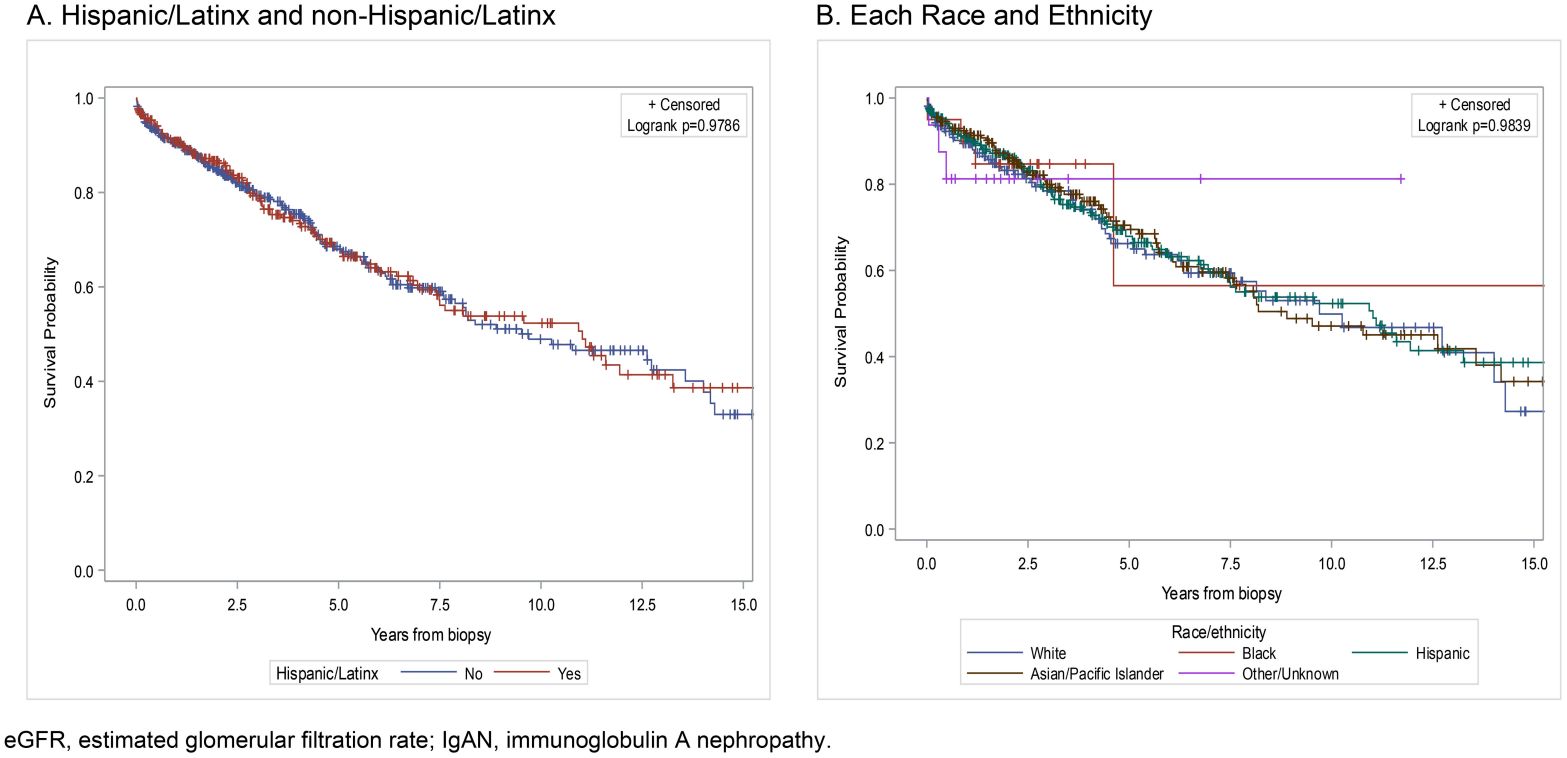
Kaplan–Meier curves for the time to the composite kidney outcome (≥50% eGFR decline or kidney failure) by race and ethnicity in individuals with biopsy-proven IgAN. **(A)** Hispanic/Latinx and non-Hispanic/Latinx. **(B)** Each race and ethnicity. *eGFR*, estimated glomerular filtration rate; *IgAN*, immunoglobulin A nephropathy.

The composite endpoint of ≥50% decline in eGFR or kidney failure occurred at an incidence (95%CI) of 73.3 events (59.2–90.8) per 1,000 patient-years among the Hispanic/Latinx population. The Kaplan–Meier curves for the time to the composite endpoint are shown for the Hispanic/Latinx and non-Hispanic/Latinx patients in **Figure 2A**. The time to composite kidney event and the age at event for the Hispanic/Latinx group were similar to those for the Asian/Pacific Islanders (**Figure 2B**). The median (IQR) time to event for the composite endpoint was 2.8 years (0.9–5.5 years) for the Hispanic/Latinx population and was 2.9 years (1.3–5.7 years) for the Asian/Pacific Islander population, while the median (IQR) age at event was 46.1 years (39.9–54.5 years) for the Hispanic/Latinx group and was 47.5 years (39.8–60.3 years) for the Asian/Pacific Islander group.

## Discussion

In summary, Hispanic/Latinx patients with IgAN in Southern California already had significant CKD at the time of their diagnosis by kidney biopsy, as demonstrated by the low baseline eGFR and the high proteinuria concentrations. Nearly one-third of the Hispanics/Latinx patients met the threshold for rapid eGFR decline. The rate of decline in kidney function was numerically higher for Hispanic/Latinx patients with IgAN compared with the other racial groups, although the differences among the racial/ethnic populations did not reach statistical significance. The slope of eGFR decline in the Hispanic/Latinx patients was closest to that of the Asian/Pacific Islander patients, who have a known elevated risk of IgAN progression (4, 6).

In terms of the composite endpoint of ≥50% decrease in eGFR or kidney failure, the clinical progression profiles of the Hispanic/Latinx and Asian/Pacific Islander populations were also similar, with a median time to event of ≤3 years from diagnosis and similar ages at the event. This finding for the composite endpoint is consistent with the reported results for kidney failure alone (11). The median (IQR) time to kidney failure was 2.8 years (0.9–5.5 years) for Hispanics/Latinx and was 3.0 years (1.5–6.3 years) for Asians/Pacific Islanders. The median (IQR) age at kidney failure was 46.1 years (39.9–54.5 years) for Hispanics/Latinx and was 47.1 years (41.8–60.9 years) for Asians/Pacific Islanders (11).

We analyzed a large, biopsy-proven IgAN cohort representative of a racially and ethnically diverse Southern California population. The use of KPSC electronic health records enabled analysis of comprehensive patient data from an integrated delivery network. Methodological limitations that may confound the interpretation of our findings include reliance on real-world practice data in which the management of IgAN may have differed among healthcare providers. Due to the limited availability of data, we were unable to address the potential for immortal time bias and delayed biopsy, which could affect the eGFR decline and rate estimates. The Hispanic/Latinx population of KPSC enrollees may not be representative of other Hispanic/Latinx populations in the United States or Latin American countries. Furthermore, the data on race/ethnicity were collected from self-identified or provider-reported information, which may be subjective and variable, and information on diverse ancestries (e.g., African and European) among the Hispanic/Latinx patients was not available. Hispanic/Latinx individuals are at risk of experiencing poorer control and management of chronic diseases; however, social determinants of health were not assessed (15). In this study, the Hispanic/Latinx patients with IgAN did have nearly identical rates of immunosuppression treatment (40.9% *vs.* 40.8%) and renin–angiotensin system blocker use (68% *vs*. 70%) compared with all IgAN patients within KPSC. The fact that approximately 30% of patients across racial and ethnic groups did not receive renin–angiotensin aldosterone inhibitors, the standard of care for patients with IgAN, suggests possible undertreatment (16). However, the presence of contraindications to the use of these agents, such as hypotension, hyperkalemia, and intolerance, was not captured in the study population. Overall, our findings indicate a clear need for earlier screening and detection and, in appropriate cases, early disease management in this population. The risk of kidney disease progression among Hispanic/Latinx patients is concerningly high and may be similar to the elevated risk observed in Asian/Pacific Islander individuals. However, due to the small sample size of our study cohort, it is unclear whether the similarities observed between the Hispanic/Latinx and Asian patients with IgAN in our study are generalizable to the US population. Additional research is needed to determine whether the findings here apply to Hispanic/Latinx patients in other geographic areas.

## Data Availability

The original contributions presented in the study are included in the article/**Supplementary Material**. Further inquiries can be directed to the corresponding author.
