# Supplementary material for: Progression of immunoglobulin A nephropathy (IgAN) in a Hispanic/Latinx population in the United States
Source: Front Nephrol. 2026 Jan 9;5:1744454. doi: 10.3389/fneph.2025.1744454 (PMC12827094; doi:10.3389/fneph.2025.1744454)
Supplement: Supplementary file 1 [file DataSheet1.pdf]

Supplemental Table 1. Baseline characteristics of cohort with immunoglobulin A nephropathy

	Asian/Pacific Islander (n=201)	Black (n=20)	Hispanic (n=259)	White (n=159)	Other/Unknown (n=16)	Total (N=655)	*P*-Value[f]
**Age in years at index**							0.43
Mean (SD)	45.1 (14.0)	45.3 (17.4)	44.7 (14.0)	47.2 (16.4)	39.8 (9.2)	45.4 (14.6)	
18 to 29, n (%)	24 (11.9%)	4 (20.0%)	42 (16.2%)	31 (19.5%)	1 (6.3%)	102 (15.6%)	
30 to 44, n (%)	88 (43.8%)	6 (30.0%)	95 (36.7%)	44 (27.7%)	10 (62.5%)	243 (37.1%)	
45 to 64, n (%)	65 (32.3%)	7 (35.0%)	95 (36.7%)	59 (37.1%)	5 (31.3%)	231 (35.3%)	
≥65, n (%)	24 (11.9%)	3 (15.0%)	27 (10.4%)	25 (15.7%)	0 (0.0%)	79 (12.1%)	
**Sex, n (%)**							<.01
Female	118 (58.7%)	8 (40.0%)	128 (49.4%)	51 (32.1%)	6 (37.5%)	311 (47.5%)	
Male	83 (41.3%)	12 (60.0%)	131 (50.6%)	108 (67.9%)	10 (62.5%)	344 (52.5%)	
**SBP[a] in mmHg, mean (SD)**	126.5 (14.8)	134.4 (16.4)	129.2 (13.0)	130.2 (13.6)	133.1 (8.3)	128.8 (13.8)	0.02
**DBP[a] in mmHg, mean (SD)**	77.3 (9.3)	80.0 (11.3)	76.7 (8.9)	76.3 (10.3)	80.6 (7.7)	77.0 (9.4)	0.40
**BMI in kg/m^2, mean (SD)**	26.3 (4.9)	28.4 (6.3)	30.9 (6.6)	29.5 (6.4)	29.5 (6.1)	29.0 (6.4)	<.01
**BMI >30 kg/m^2, n (%)**							<.01
No	153 (76.1%)	10 (50.0%)	128 (49.4%)	84 (52.8%)	10 (62.5%)	385 (58.8%)	
Yes	35 (17.4%)	6 (30.0%)	110 (42.5%)	62 (39.0%)	5 (31.3%)	218 (33.3%)	
Unknown	13 (6.5%)	4 (20.0%)	21 (8.1%)	13 (8.2%)	1 (6.3%)	52 (7.9%)	
**Smoking status, n (%)**							0.09
Non-smoker	149 (74.1%)	12 (60.0%)	172 (66.4%)	99 (62.3%)	14 (87.5%)	446 (68.1%)	
Quit smoking	43 (21.4%)	5 (25.0%)	74 (28.6%)	51 (32.1%)	2 (12.5%)	175 (26.7%)	
Current smoker	7 (3.5%)	1 (5.0%)	9 (3.5%)	7 (4.4%)	0 (0.0%)	24 (3.7%)	
Unknown	2 (1.0%)	2 (10.0%)	4 (1.5%)	2 (1.3%)	0 (0.0%)	10 (1.5%)	
**Elixhauser Comorbidity Index,[b] mean (SD)**	2.5 (1.8)	2.3 (2.6)	2.9 (1.9)	3.4 (2.8)	2.2 (1.4)	2.9 (2.2)	<.01
**Hypertension,[b]** n (%)	132 (65.7%)	11 (55.0%)	164 (63.3%)	101 (63.5%)	10 (62.5%)	418 (63.8%)	0.91
**Diabetes,[b]** n (%)	33 (16.4%)	0 (0.0%)	37 (14.3%)	18 (11.3%)	1 (6.3%)	89 (13.6%)	0.19

**Coronary artery disease**,[b] n (%)	8 (4.0%)	0 (0.0%)	4 (1.5%)	10 (6.3%)	0 (0.0%)	22 (3.4%)	0.08
**Heart failure**,[b] n (%)	1 (0.5%)	0 (0.0%)	5 (1.9%)	6 (3.8%)	1 (6.3%)	13 (2.0%)	0.15
**Stroke**,[b] n (%)	3 (1.5%)	0 (0.0%)	1 (0.4%)	4 (2.5%)	0 (0.0%)	8 (1.2%)	0.34
**Myocardial infarction**,[b] n (%)	1 (0.5%)	0 (0.0%)	4 (1.5%)	2 (1.3%)	0 (0.0%)	7 (1.1%)	0.74
**Atrial fibrillation**,[b] n (%)	8 (4.0%)	0 (0.0%)	5 (1.9%)	11 (6.9%)	0 (0.0%)	24 (3.7%)	0.08
**Peptic ulcer**,[b] n (%)	5 (2.5%)	0 (0.0%)	1 (0.4%)	2 (1.3%)	0 (0.0%)	8 (1.2%)	0.29
**Hematuria**,[b] n (%)	92 (45.8%)	14 (70.0%)	123 (47.5%)	90 (56.6%)	8 (50.0%)	327 (49.9%)	0.13
**Baseline eGFR[a,c] in ml/min/1.73m^2**							
Mean (SD)	61.4 (29.3)	58.5 (24.9)	62.3 (31.6)	54.1 (26.3)	62.1 (28.5)	59.9 (29.5)	
Median (IQR)	54.6 (37.5, 82.5)	63.3 (39.7, 79.5)	56.0 (36.6, 82.8)	49.1 (33.6, 69.6)	59.6 (46.3, 78.4)	53.8 (36.5, 79.6)	0.11
≥90, n (%)	37 (18.4%)	2 (10.0%)	56 (21.6%)	22 (13.8%)	4 (25.0%)	121 (18.5%)	
60-89, n (%)	53 (26.4%)	10 (50.0%)	61 (23.6%)	33 (20.8%)	4 (25.0%)	161 (24.6%)	
45-59, n (%)	47 (23.4%)	2 (10.0%)	42 (16.2%)	37 (23.3%)	4 (25.0%)	132 (20.2%)	
30-44, n (%)	36 (17.9%)	2 (10.0%)	65 (25.1%)	38 (23.9%)	2 (12.5%)	143 (21.8%)	
15-29, n (%)	28 (13.9%)	4 (20.0%)	35 (13.5%)	29 (18.2%)	2 (12.5%)	98 (15.0%)	
**Treatment with immunosuppressive agents,[d] n (%)**	83 (41.3%)	11 (55.0%)	106 (40.9%)	61 (38.4%)	6 (37.5%)	267 (40.8%)	0.71
**ACEi**,[b] n (%)	86 (42.8%)	5 (25.0%)	125 (48.3%)	74 (46.5%)	6 (37.5%)	296 (45.2%)	0.27
**ARB,**[b] n (%)	74 (36.8%)	3 (15.0%)	51 (19.7%)	27 (17.0%)	6 (37.5%)	161 (24.6%)	<.01
**GLP-1,**[b] n (%)	0 (0.0%)	0 (0.0%)	0 (0.0%)	0 (0.0%)	1 (6.3%)	1 (0.2%)	0.12
**SGLT-2i,**[b] n (%)	0 (0.0%)	0 (0.0%)	1 (0.4%)	1 (0.6%)	0 (0.0%)	2 (0.3%)	0.75
**Baseline UPCR[e] in g/g**							
Mean (SD)	2.7 (2.5)	1.6 (1.9)	2.5 (2.4)	2.2 (2.2)	2.3 (2.3)	2.5 (2.4)	
Median (IQR)	2.0 (1.0, 3.7)	0.8 (0.3, 2.0)	1.8 (1.0, 3.2)	1.6 (0.7, 3.1)	1.5 (0.5, 3.0)	1.8 (0.9, 3.3)	0.02
<0.5, n (%)	18 (9.0%)	6 (30.0%)	32 (12.4%)	24 (15.1%)	4 (25.0%)	84 (12.8%)	
0.5-<1, n (%)	26 (12.9%)	4 (20.0%)	27 (10.4%)	23 (14.5%)	2 (12.5%)	82 (12.5%)	

1-2, n (%)	57 (28.4%)	2 (10.0%)	74 (28.6%)	46 (28.9%)	3 (18.8%)	182 (27.8%)
>2, n (%)	95 (47.3%)	5 (25.0%)	111 (42.9%)	54 (34.0%)	6 (37.5%)	271 (41.4%)
Unknown, n (%)	5 (2.5%)	3 (15.0%)	15 (5.8%)	12 (7.5%)	1 (6.3%)	36 (5.5%)

[a]Based on the most recent record within 1 year prior to or as of kidney biopsy.

[b]Comorbidity and medication were based on data within 1 year prior to or as of kidney biopsy.

[c]Measurements in the inpatient setting were excluded.

[d]With immunosuppressive agents during 4 weeks prior to and 1 year after kidney biopsy.

[e]Record measured closest to kidney biopsy during 1 year before and 30 days after biopsy was retained. Urine albumin-creatinine ratio and total urine protein within 24 hours were converted to UPCR by dividing by 0.7 and 1000, respectively.

[f]For comparison of characteristics among the five racial/ethnic groups (Asian/Pacific Islander, Black, Hispanic/Latinx, White, and other/unknown).

ACEi, angiotensin converting enzyme inhibitor; ARB, angiotensin receptor blocker; BMI, body mass index; DBP, diastolic blood pressure; eGFR, estimated glomerular filtration rate; GLP-1, glucagon-like peptide 1 agonist; SBP, systolic blood pressure; SGLT-2i, sodium glucose costransporter-2 inhibitor; UPCR, urine protein creatinine ratio.
